# Role of cardiovascular health factors in mediating social inequalities in the incidence of dementia in the UK: two prospective, population-based cohort studies

**DOI:** 10.1016/j.eclinm.2024.102539

**Published:** 2024-03-14

**Authors:** Frank C.T. van der Heide, Linda Valeri, Aline Dugravot, Ian Danilevicz, Benjamin Landre, Mika Kivimaki, Séverine Sabia, Archana Singh-Manoux

**Affiliations:** a Université Paris Cité, Inserm U1153, Epidemiology of Ageing and Neurodegenerative Diseases, Paris, France; b Department of Biostatistics, Columbia University Mailman School of Public Health, New York, NY, USA; c Department of Epidemiology, Harvard T.H. Chan School of Public Health, Boston, MA, USA; d Faculty of Brain Sciences, University College London, UK; e Clinicum, Faculty of Medicine, University of Helsinki, Helsinki, Finland

**Keywords:** Social economic inequalities, Dementia, Counterfactual mediation, Life's Essential 8 score

## Abstract

**Background:**

The contribution of modifiable risk factors to social inequalities in dementia, observed in longitudinal studies, remains unclear. We aimed to quantify the role of cardiovascular health factors, assessed using Life's Essential 8 (LE8) score, in mediating social inequalities in incidence of dementia and, for comparison, in incidence of stroke, coronary heart disease, and mortality.

**Methods:**

In this prospective, population-based cohort study, we collected data from the UK Whitehall II Study and UK Biobank databases. Participants were included if data were available on SEP, outcomes and LE8 (smoking, physical activity, diet, body mass index, blood pressure, fasting blood glucose, lipid levels, sleep duration). The primary outcome was incident dementia and secondary outcomes were stroke, coronary heart disease, and mortality. Outcomes were derived from electronic healthcare records. Socioeconomic position (SEP) was measured by occupation in Whitehall II and education in UK Biobank. Counterfactual mediation analysis was used to quantify the extent to which LE8 score explained the associations of SEP with all outcomes. Analyses involved Cox regression, accelerated failure time models, and linear regression; and were adjusted for age, sex, and ethnicity.

**Findings:**

Between 10.09.1985 and 29.03.1988, a total of 9688 participants (mean age ± SD 44.9 ± 6.0; 67% men) from the Whitehall II study, and between 19.12.2006 and 01.10.2010, 278,215 participants (mean age ± SD 56.0 ± 8.1; 47% men) from the UK Biobank were included. There were 606 and 4649 incident dementia cases over a median (interquartile range) follow-up of 31.7 (31.1–32.7) and 13.5 (12.7–14.1) years respectively in Whitehall II and UK Biobank. In Whitehall II, the hazard ratio was 1.85 [95% CI 1.42, 2.32] for the total effect of SEP on dementia and 1.20 [1.12, 1.28] for the indirect effect via the LE8, the proportion mediated being 36%. In UK Biobank, the total effect of SEP on dementia was 1.65 [1.54, 1.78]; the indirect effect was 1.11 [1.09, 1.12], and the proportion mediated was 24%. The proportions mediated for stroke, coronary heart disease, and mortality were higher, ranging between 34% and 63% in Whitehall II and between 36% and 50% in UK Biobank.

**Interpretation:**

In two well-characterised cohort studies, up to one third of the social inequalities in incidence of dementia was attributable to cardiovascular health factors. Promotion of cardiovascular health in midlife may contribute to reducing social inequalities in risk of dementia, in addition to cardiovascular diseases and all-cause mortality. This study used adult measures of SEP, further research is warranted using lifecourse measures of SEP.

**Funding:**

10.13039/100000002NIH (RF1AG062553)

**Research in context**

**Evidence before this study**

We searched PubMed up to 1st of November 2023 to identify all available evidence on mediation of the association between social economic position (occupational position, education, income) and outcomes (dementia, stroke, coronary heart disease, and mortality) by modifiable cardiovascular health factors (smoking, physical activity, healthy diet, body mass index, blood pressure, fasting blood glucose, blood lipids, sleep duration). Almost all studies investigated modifiable health factors in late-life, rather in midlife, whilst targeting health factors in midlife is likely more beneficial. No studies considered all health factors included in Life's Essential 8 score; and the relatively short follow-up in almost all studies precludes conclusions on exposure to health factors prior to the long (15–20 years) preclinical phase of dementia.

**Added value of this study**

Using two longitudinal studies, Whitehall II and UK Biobank with a median follow-up of 31.7 and 13.5 years respectively, we show that a third of the excess risk of dementia in socially disadvantaged groups can be explained by Life's Essential 8 score. The present study is the largest to date and covers the longest follow-up period. Novel is the quantitative insight into the contributions of a comprehensive cluster of cardiovascular health factors in midlife, prior even to the preclinical phase of dementia. As expected, the contribution of Life's Essential 8 to social inequalities in cardiovascular diseases and total mortality was larger and similar to that in the literature, providing confidence in the findings on dementia.

**Implications of all the available evidence**

Promotion of cardiovascular health in midlife may contribute to reducing social inequalities in risk of dementia, in addition to cardiovascular diseases and all-cause mortality. These findings are particularly relevant for countries that are developing nationwide action plans for the prevention of dementia. This study used adult measures of SEP, further research is warranted using lifecourse measures of SEP.

## Introduction

The number of people living with dementia is increasing worldwide, and is projected to reach ∼80 million in 2030 and ∼150 million in 2050.^1^ Besides research on disease-modifying treatments, there is increased attention on prevention by targeting potentially modifiable risk factors.^2^ The long preclinical phase of dementia, up to 15–20 years,^3^ suggests that attempts to slow and/or prevent the onset of dementia via modification of risk factors needs to target midlife.^4^ As the risk of dementia is higher in socially disadvantaged groups, estimated to be two-fold higher in a recent meta-analysis using education or occupation,^5^ it is possible that disadvantaged groups derive greater benefit from modification of risk factors.

The role of lifestyle and cardiometabolic factors in mediating the association between socioeconomic factors and dementia has been examined in recent studies.^6^, ^7^, ^8^, ^9^ We note important limitations in these studies. Most are based on measurement of risk factors at older ages or have relatively short follow-ups (<12 years), making the results subject to reverse causation bias due to the long preclinical period of dementia. One study found 25% of the association between education and dementia to be mediated by four mid-life vascular risk factors considered individually.^9^ An alternative approach is to consider the overall impact of multiple factors in a risk score to better consider the clustering of risk factors.^10^ Furthermore, achieving an improvement in a score might be more feasible for individuals than optimal targets for specific risk factors. The American Heart Association's Life's Essential 8 score is one such tool, with a prevention perspective for promoting ideal cardiovascular health to improve a range of health outcomes.^11^ The score is calculated from eight components to encourage: eating better, being physically active, not smoking, healthy sleep, controlling cholesterol, and managing weight, blood sugar and blood pressure.^11^

The primary aim of our study is to examine the extent to which Life's Essential 8 score^11^ mediates the association between socioeconomic position (SEP) and incident dementia using a median 31.7-year follow-up in the Whitehall II study, with replication in the UK Biobank study. For comparison, we also examined this research question with secondary outcomes: stroke, coronary heart disease, and all-cause mortality.

## Methods

### Study population and design

We adhered to the Strengthening the Reporting of Observational Studies in Epidemiology (STROBE) Statement.^12^ The Whitehall II study is an observational cohort study on 10,308 participants.^13^ Eligible for participation were all men and women, aged 35–55 years old, working in the London offices of twenty civil-service departments in 1985.^13^ The response rate to the written invitation was 73%.^13^ Baseline measurements took place between 10.09.1985 and 29.03.1988; and follow-up clinical examinations have taken place approximately every 4–5 years.^13^ All participants gave written consent for participation. The Whitehall II study received approval from the University College London Hospital Committee on the Ethics of Human Research (reference number 85/0938).^13^

UK Biobank is an observational, population-based cohort study on 502,371 participants.^14^ Eligible for participation were all individuals aged between 40 and 69 years, registered with the UK National Health Service.^14^ Members of the target population were invited to participate by letter. The response rate was ∼6%.^14^ Baseline measurements took place between 19.12.2006 and 01.10.2010.^14^ UK Biobank received approval from the National Information Governance Board for Health and Social Care and the National Health Service North West Centre for Research Ethics Committee (reference number 11/NW/0382).^14^ All participants gave written consent for participation. This research has been conducted using the UK Biobank Resource under application number 96856.

### Assessment of SEP

SEP was measured using grade of employment at baseline (1985–88) in the Whitehall II Study. Employment grade is a three-level variable ranging from high (administrative grade) to low (clerical and support staff, e.g., messengers, porters, telephonists, typists). The measure is attributed to everyone in the civil service and is a comprehensive marker, reflecting education, salary, social status, and level of responsibility at work.^13^

SEP was measured using self-reported educational level in UK biobank. We chose to use educational level because data on this measure were available for a considerably higher number of participants (n = 497,724) than for occupation (n = 324,951). We categorized education as follows: high (college/university degree, other professional qualification - e.g., nursing, teaching), intermediate (lower secondary, second/final stage of secondary education, vocational qualifications) and low (lower than the previous mentioned categories). In sensitivity analysis, occupation instead of education was used as the SEP marker (details in Supplemental Methods). In sensitivity analysis, occupation instead of education was used as a marker of SEP in UK Biobank (details in Supplemental Methods).

### Assessment of Life's Essential 8 score

Life's Essential 8 score (0–800 points, higher scores signify better cardiovascular health) was calculated by summing scores for eight risk factors using the original scoring scheme (smoking, physical activity, healthy diet, body mass index, blood pressure, fasting blood glucose, non-high density lipoprotein (non-HDL) cholesterol, sleep duration)^11^; details are provided in the Supplemental Methods and in Table S1. In the Whitehall II study all components were drawn from the wave when participants were closest to 50 years using data from 1985 (age range 35–55 years), 1991 (40–64 years), 1997 (45–69 years), 2002 (50–74 years) and 2007 (55–79 years), yielding a range between 34.7 and 59.9 years. In UK Biobank Life's Essential 8 score was also measured using the original scoring scheme, at study baseline (2006–2010; individuals were aged 40–69 years).

### Covariates

The covariates included age at baseline (years), sex (male/female), and ethnicity (white, non-white) and apolipoprotein E4 (APOE4).

### Dementia (primary outcome)

All-cause dementia in both cohorts was identified using linkage to electronic health records (EHR) using ICD-10 codes F00-F03, F05.1, G30, and G31. The sensitivity and specificity for case identification using the national Hospital Episodes Statistics (HES) data, respectively, are 78.0% and 92.0% for all-cause dementia.^15^ In addition, sensitivity is likely to be further improved due to additional use of the national mortality register data (both cohorts); and the Mental Health Services Data Set (only Whitehall II study), a national database that contains information on dementia for persons in contact with mental health services in hospitals, outpatient clinics, and the community.^16^ The date of incident dementia was set at the first date at which a diagnosis was identified using all health records. In UK Biobank study data on subtype of dementia (Alzheimer's disease and vascular dementia) were also available, and were used in additional analyses.

### Stroke, coronary heart disease, and mortality (secondary outcomes)

In both studies stroke was identified via linkage to EHR using ICD-10 codes I60, I61, I63, I64^17^; and coronary heart disease using ICD-9 codes 410–414 and 429; and ICD-10 codes I20–I25.^17^ The sensitivity and specificity for case identification are 71.0% and 100.0% for stroke, and 70.0% and 96.0% for coronary heart disease.^15^ The date of incident stroke and coronary heart disease was set at the first date at which a diagnosis was identified using all health records. Data on mortality were drawn from the national mortality register.^16^

### Statistical analysis

The start of the follow-up was from baseline resulting in a median follow-up of 31.7 and 13.5 years in Whitehall II and UK Biobank, respectively. Participants with prevalent dementia, stroke, or coronary heart disease at measurement of Life's Essential 8 score, and in Whitehall II participants who entered the study after age 60.0 years were excluded from the analyses.

**Fig. 1:**
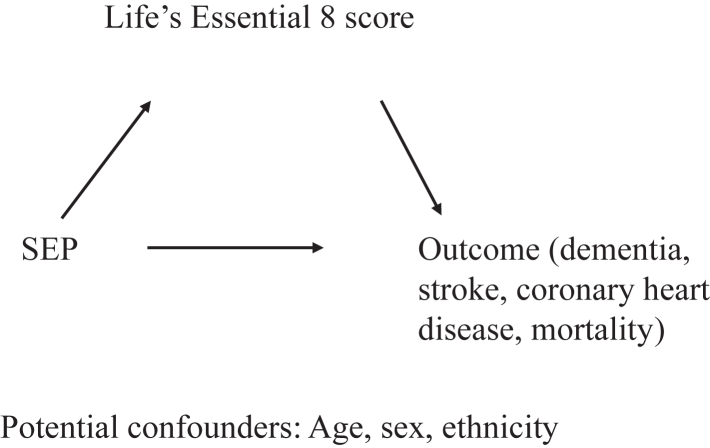
**Counterfactual mediation framework.** Directed Acyclic Graph (DAG) for the analysis of the contribution of modifiable risk factors, estimated from the Life's Essential 8 score, to the association between SEP and dementia, and secondary outcomes (stroke, coronary heart disease, and mortality). *Abbreviation* SEP: socioeconomic position (measured using occupational position in Whitehall II and using educational level in UK Biobank).

We used counterfactual mediation analysis to quantify the extent to which Life's Essential 8 score explained the associations of SEP with incident dementia (primary outcome), and secondary outcomes (stroke, coronary heart disease, and mortality) in both cohort studies (separate analyses), as shown in Fig. 1. We conducted the analyses in R (R version 4.0.3, R Foundation for Statistical Computing, Vienna, Austria) using the *CMAverse* package.^18^

The counterfactual framework allows for the quantification of the indirect effect (i.e., the association of SEP with an outcome that is mediated by Life's Essential 8 score), the direct effect (i.e., the association of SEP with the outcome that is not mediated by Life's Essential 8 score), the total effect (the direct and indirect effect), and the percentage mediation (reflecting the proportion of the total effect mediated by the indirect effect).^19^ Effects were expressed on the hazard ratio scale. We use the term ‘effects’ instead of ‘associations’ because this is a commonly used term in the field of mediation. The term ‘effect’ does not imply that causality can be inferred from our findings.

SEP was used as a continuous variable, ranging from 0 (high SEP) to 0.5 (middle SEP) and 1 (low SEP), so that a change of one point (from 0 to 1) reflects risk in low compared to high SEP group.^20^ We used Cox regression analysis with age at time scale to calculate the associations of SEP and Life's Essential 8 score with dementia and secondary outcomes.^19^ We verified the proportional hazards assumption was not violated^21^ and that were no exposure-mediator interactions.^19^ Date of censoring was set at the date of outcome (applicable for dementia, stroke, coronary heart disease), end of follow-up, or date of death, whichever occurred first. Last date at which data on outcomes were available in Whitehall II was 31/03/2019. Last dates at which data on outcomes were available in UK Biobank were: 31/10/2022 (UK); 28/02/2018 (Wales); and 31/07/2021 (Scotland).

For outcomes that were not rare (i.e., incidence ≥10%), we used an accelerated failure time model to analyse associations (generating survival time ratios).^19^ In the present study, we used this approach for coronary heart disease and mortality (incidence 20% and 21%, respectively in Whitehall II; in UK Biobank: 8% and 7%, respectively). To facilitate the interpretation of results we inverted the direction of the survival time ratio's (generating Failure Time Ratios) so that results for coronary heart disease and mortality were expressed in the same direction as results for dementia and stroke.

For all analyses, we adjusted for age at measurement of SEP, sex, and ethnicity; used bootstrapping (n = 200 samples) to calculate confidence intervals^18^; and used inverse probability weights to account for potential selection bias.^22^ Further details on the counterfactual framework, the evaluation of assumptions, the accelerated failure time model, and the calculation of weights are provided in the Supplemental Methods and in Table S2.

#### Analyses of interaction

We examined whether the association of SEP with outcomes differed as a function of sex by using an interaction term between SEP and sex in the association of SEP with Life's Essential 8 score; in the association of Life's Essential 8 score with outcomes; and in the association of SEP with outcomes. We also evaluated interaction by ethnicity in associations of SEP and Life's Essential 8 Score with incident dementia; and SEP with Life's Essential 8 Score.

#### Additional analyses

One, we undertook further counterfactual mediation analyses using subtypes of dementia as the outcome (i.e., Alzheimer's disease and vascular dementia) in UK Biobank. Two, we used the simulation extrapolation (SIMEX) approach to obtain quantitative insight into the impact of measurement error of the mediator.^23^ This method calculates counterfactual mediation effect estimates when varying levels of measurement error in the mediator are simulated.^23^ Three, we examined the potential impact of missing confounders using mediational E-values, that indicate the minimum strength of association that an unmeasured confounder would need to have with both the mediator and the outcome to explain away the observed association.^24^ An online calculator was used for estimation of mediational E-values, it used the following formula: HR^observed^ + √ (HR^observed^ × [HR^observed^ − 1]).^24^^,^^25^ Four, as coronary heart disease and mortality were not strictly rare outcomes in UK Biobank (i.e., 5–10% incident cases instead of ≥10%), we repeated the analyses in UK Biobank using Cox regression instead of the accelerated failure time model. Five, we calculated population attributable risk by Life's Essential 8 score for incident dementia according to level of SEP. We dichotomized Life's Essential 8 score based on the median score in the total population. Six, we additionally adjusted for APOE4 in analyses with dementia as outcome. Seven, to obtain insight into the role of mediation by individual health factors, we evaluated to which extent results changed when one of the health variables was omitted from Life's Essential 8 score. Eight, we repeated analyses in UK Biobank using occupational level as a measure of SEP instead of educational level.

For all analyses, P-value <0.05 was considered statistically significant.

### Role of the funding source

The funder of the study had no role in study design, data collection, data analysis, data interpretation, or writing of the report. FCTvdH and AD had access to the dataset. ASM and SS had the final responsibility for the decision to submit for publication.

## Results

**Fig. 2:**
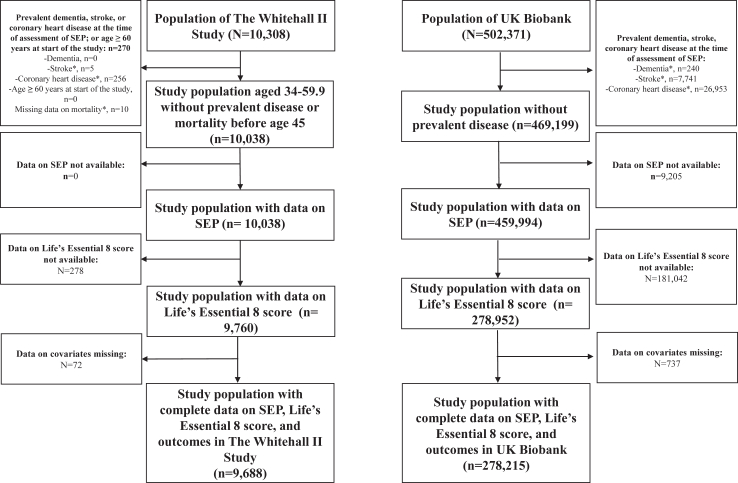
**Flow-chart for participants included in the analyses in Whitehall II and UK Biobank cohort studies.** ∗ not mutually exclusive. *Abbreviation* SEP: socioeconomic position.

Fig. 2, a flow-chart, shows the participants included in the analyses in Whitehall II and UK Biobank. The analyses in Whitehall II were on 9688 participants (mean age at baseline ± SD 44.9 ± 6.0 years; 67% men) and mean age ± SD at assessment of Life's Essential 8 score was 49.5 ± 4.3 years. The median follow-up (interquartile range) was 31.7 (31.1, 32.7) years, and there were 606, 463, and 1965 incident cases of dementia, stroke, and coronary heart disease, respectively; and 2001 deaths. The UK Biobank analyses were based on 278,215 participants (mean age at baseline ± SD 56.0 ± 8.1 years; 47% men); the median follow-up (interquartile range) was 13.5 (12.7–14.1) years, and there were 4649, 5840, and 21,601 incident cases of dementia, stroke, and coronary heart disease, respectively; and 19,089 deaths. Characteristics of participants in both studies are shown in Table 1.

**Table 1:** Baseline characteristics of participants in Whitehall II and UK Biobank studies as a function of dementia, stroke, coronary heart disease, and mortality.

	Total	Primary outcome		Secondary Outcomes					
		Dementia		Stroke		Coronary heart disease		Mortality	
		No	Yes	No	Yes	No	Yes	No	Yes
**Whitehall II Study**	N = 9688	N = 9082	N = 606	N = 9225	N = 463	N = 7723	N = 1965	N = 7687	N = 2001
Age (years), M (SD)	44.85 (6.01)	44.52 (5.93)	49.80 (4.90)	44.70 (5.98)	47.84 (5.79)	44.20 (5.92)	47.38 (5.67)	43.91 (5.74)	48.40 (5.68)
Men, n (%)	6511 (67)	6156 (68)	355 (59)	6196 (67)	315 (68)	5082 (66)	1429 (73)	5211 (68)	1300 (65)
Women, n (%)	3177 (33)	2926 (32)	251 (41)	3059 (33)	148 (32)	2641 (34)	536 (27)	2476 (32)	701 (35)
Occupational position, n (%)									
1 High	2883 (30)	2728 (30)	155 (26)	2750 (30)	133 (29)	2312 (30)	571 (29)	2340 (30)	543 (27)
2	4687 (48)	4450 (49)	237 (39)	4486 (49)	201 (43)	3782 (49)	905 (46)	3798 (49)	889 (44)
3 Low	2118 (22)	1904 (21)	214 (35)	1989 (22)	129 (28)	2118 (22)	489 (25)	1549 (20)	569 (28)
Non-white Ethnicity, n (%)	942 (9.7)	860 (9.5)	82 (14)	880 (9.5)	62 (13)	942 (9.7)	288 (15)	736 (9.6)	206 (10)
Life's Essential 8, M (SD)	570 (101)	572 (101)	543 (100)	571 (100)	552 (111)	570 (101)	540 (102)	579 (98)	537 (104)
Median follow-up (IQR)	31.7 (31.1, 32.7)	31.8 (31.2, 32.7)	27.5 (24.6, 30.2)	31.8 (31.2, 32.7)	24.1 (18.1, 28.5)	31.5 (26.4, 32.4)	19.6 (12.7, 25.8)	32.1 (31.5, 32.8)	24.1 (17.2, 28.5)
**UK Biobank**	N = 278,215	N = 273,566	N = 4649	N = 272,375	N = 5840	N = 256,614	N = 21,601	N = 259,126	N = 19,089
Age (years), M (SD)	55.96 (8.10)	55.83 (8.07)	63.66 (5.28)	55.86 (8.09)	60.84 (6.80)	55.64 (8.10)	59.88 (6.98)	55.58 (8.06)	61.20 (6.61)
Men, n (%)	129,567 (47)	127,086 (46)	2481 (53)	126,212 (46)	3355 (57)	115,542 (45)	14,025 (65)	118,319 (46)	11,248 (59)
Women, n (%)	148,648 (53)	146,480 (54)	2168 (47)	146,163 (54)	2485 (43)	141,072 (55)	7576 (35)	140,807 (54)	7841 (41)
Educational level, n (%)									
1 High	142,532 (51)	140,699 (51)	1833 (39)	139,999 (51)	2533 (43)	133,042 (52)	9490 (44)	134,401 (52)	8131 (43)
2	98,856 (36)	97,375 (36)	1481 (32)	96,823 (36)	2033 (35)	91,436 (36)	7420 (34)	92,533 (36)	6323 (33)
3 Low	36,827 (13)	35,492 (13)	1335 (29)	35,553 (13)	1274 (22)	32,136 (13)	4691 (22)	32,192 (12)	4635 (24)
Non-white Ethnicity, n (%)	23,920 (8.6)	23,587 (8.6)	333 (7.2)	23,482 (8.6)	438 (7.5)	22,232 (8.7)	1688 (7.8)	22,546 (8.7)	1374 (7.2)
Life's Essential 8, M (SD)	615 (85)	615 (85)	588 (86)	616 (84)	581 (88)	618 (83)	574 (87)	618 (83)	576 (91)
Median follow-up (IQR)	13.5 (12.7, 14.1)	13.5 (12.7, 14.1)	10.7 (8.1, 12.4)	13.5 (12.7, 14.1)	9.0 (5.3, 11.6)	13.5 (12.7,14.1)	7.6 (4.2, 10.6)	13.6 (12.9, 14.1)	9.2 (5.8, 11.7)

*Abbreviations* M: Mean; SD: standard deviation; IQR: interquartile range.

Figs. S1 and S2 show plots of Life's Essential 8 score across SEP categories in Whitehall II and UK Biobank. Table S3 shows incidence rate of outcomes according to SEP (per 1000 person-years). In both cohorts there was a social gradient, where higher SEP was associated with a higher Life's Essential 8 score and a lower incidence of all outcomes.

Table 2 shows SEP to be more strongly associated with Life's Essential 8 score in Whitehall II than in UK Biobank (beta [95% CI] for low versus high SEP, −73 [−80, −67] points and −43 [−44; −42] points, respectively). The association of Life's Essential 8 score with dementia was similar in Whitehall II and UK Biobank (estimate per 100 points higher score, HR [95% CI] 0.78 [0.72, 0.85] and 0.80 [0.77, 0.83]). The association of Life's Essential 8 score with secondary outcomes was similar in strength to the associations with dementia, but tended to be somewhat stronger in UK Biobank than in Whitehall II.

**Table 2:** Associations of SEP with Life's Essential 8 score, and that of Life's Essential 8 score with all outcomes.

	Whitehall II N = 9688	UK Biobank N = 278,215
	β (95% CI)	β (95% CI)
Low versus high SEP → Life's Essential 8 score (0–800 points)	−73 (−80, −67)	−43 (−44, −42)

	Hazard ratio (95% CI)	Hazard ratio (95% CI)
Life's Essential 8 score (per 100 points) → dementia	0·78 (0·72, 0·85)	0·80 (0·77, 0·83)
Life's Essential 8 score (per 100 points) → stroke	0·84 (0·76, 0·94)	0·70 (0·68, 0·72)

	Failure time ratio (95%)	Failure time ratio (95%)
Life's Essential 8 score (per 100 points) → coronary heart disease	0·81 (0·79, 0·83)	0·70 (0·69, 0·71)
Life's Essential 8 score (per 100 points) → mortality	0·85 (0·84, 0·88)	0·79 (0·78, 0·79)

The Hazard Ratios and Failure Time Ratios are calculated for low versus high SEP or per 100 points increase in Life's Essential 8 score. Covariates entered in all analyses in addition to SEP: age at baseline (time-scale), sex, and ethnicity. Inverse probability weights were included in the models. Hazard ratios were calculated using Cox regression and Failure Time Ratios were calculated using the accelerated failure time model.

*Abbreviations* CI: Confidence Interval; SEP: Socioeconomic Position (measured using occupational position in Whitehall II and educational level in UK Biobank).

Table 3 shows the results of counterfactual mediation analysis for all outcomes. In Whitehall II, the HR for the total effect of SEP on dementia was 1.85 [95% CI 1.42, 2.32], the direct effect was 1.54 [1.17, 1.98], the indirect effect via the Life Essential 8 score was 1.20 [1.12, 1.28], and the proportion mediation was 36% (Table 3). In UK Biobank, the total effect was 1.65 [1.54, 1.78]; the direct effect was 1.49 [1.40, 1.61]; the indirect effect was 1.11 [1.09, 1.12], and the proportion mediation was 24%.

**Table 3:** Association of SEP with dementia and secondary outcomes (stroke, coronary heart disease, and mortality): total, direct, and indirect effects and the proportion mediated by Life's Essential 8 score.

	Primary outcome	Secondary outcomes		
	Dementia	Stroke	Coronary heart disease	Mortality
Whitehall II study, n = 9688	N cases = 606	N cases = 463	N cases = 1965	N cases = 2001
Low versus high occupational position	Hazard ratio (95% CI)	Hazard ratio (95% CI)	Failure time ratio (95% CI)	Failure time ratio (95% CI)
Total effect	1·85 (1·42, 2·32)	1·52 (1·07, 2·11)	1·25 (1·15, 1·37)	1·22 (1·16, 1·30)
Direct effect	1·54 (1·17, 1·98)	1·34 (0·96, 1·95)	1·08 (0·99, 1·18)	1·10 (1·03, 1·18)
Indirect effect	1·20 (1·12, 1·28)	1·13 (1·05, 1·21)	1·16 (1·12, 1·19)	1·11 (1·09, 1·12)
**% mediation**	36%	34%	63%	50%

UK Biobank, n = 278,215	N cases = 4649	N cases = 5840	N cases = 21,601	N cases = 19,089
Low versus high educational level	Hazard ratio (95% CI)	Hazard ratio (95% CI)	Failure time ratio (95% CI)	Failure time ratio (95% CI)
Total effect	1·65 (1·52, 1·78)	1·40 (1·32, 1·50)	1·41 (1·37, 1·45)	1·30 (1·27, 1·33)
Direct effect	1·49 (1·37, 1·61)	1·20 (1·12, 1·28)	1·20 (1·18, 1·23)	1·18 (1·15, 1·19)
Indirect effect	1·11 (1·09, 1·12)	1·17 (1·15, 1·18)	1·16 (1·14, 1·18)	1·11 (1·10, 1·12)
**% mediation**	24%	50%	40%	36%

Hazard ratios and Failure Time Ratios are calculated for low versus high SEP. Covariates entered in all analyses in addition to SEP: age at baseline, sex, and ethnicity. Inverse probability weights were included in the models.

*Abbreviations* CI: Confidence Interval; SEP: Socioeconomic Position (measured using occupational position in Whitehall II and educational level in UK Biobank).

For the secondary outcomes (Table 3) in Whitehall II, the HR for the total effect of SEP on stroke, and Failure Time Ratio for coronary heart disease and mortality were 1.52 [1.07, 2.11], 1.25 [1.15, 1.37], and 1.22 [1.16, 1.30], respectively. The proportion mediated by Life Essential 8 score for stroke, coronary heart disease and mortality was 34%, 63%, and 50%, respectively. In UK Biobank, the HR for the total effect of SEP on stroke, and Failure Time Ratio for coronary heart disease and mortality were 1.40 [1.32, 1.50], 1.41 [1.37, 1.45], and 1.30 [1.27, 1.33]. The proportion mediated by Life Essential 8 score was 50%, 40%, and 36%, respectively.

### Interaction analyses

Sex did not modify associations with dementia or stroke as outcome, but did modify associations with coronary heart disease and mortality as outcomes. All P-values for interaction are presented in Table S4. Ethnicity did not modify associations under study (Table S5). Sex-stratified analyses did not show a consistent pattern for differences in mediation across cohorts. Results of stratified analyses are shown in Tables S6 and S7.

### Additional analyses

These analyses showed that, one, Life's Essential 8 score mediated 8% and 41% of the associations of SEP with incident Alzheimer's disease (n = 1362 cases) and vascular dementia (n = 536 cases), respectively in UK Biobank (Table 4). Two, the SIMEX approach with simulation for varying levels of measurement error in Life's Essential 8 score found assumptions of progressively smaller measurement error led to increase in the proportion mediation (e.g., for dementia in Whitehall II the observed proportion at 36% was 55% at the smallest measurement error simulated in Whitehall II; Table S8). The findings in UK Biobank and for other outcomes were similar. Three, E-value estimates showed that an unmeasured confounder would need to be associated with Life's Essential 8 score and dementia with a HR of 1.69 and 1.46 in Whitehall II and UK Biobank, respectively to nullify the indirect effects (Table S9). Mediational E-values for secondary outcomes were from 1.46 to 1.59 in Whitehall II and 1.36 to 1.62 in UK Biobank (Table S9). Four, mediation analysis using Cox regression instead of accelerated failure time in UK Biobank had somewhat stronger mediation by Life's Essential 8 for coronary heart disease (50% against 40% in the main analyses) and mortality (45% against 36% in the main analyses) (Table S10). Five, population attributable risk ranged between 0.19 and 0.28 in Whitehall II and between 0.10 and 0.17 in UK Biobank. Population attributable risk was higher among individuals with a low versus high SEP (Table S11). Six, we had similar findings to those shown in Table 3 when we additionally adjusted for APOE4 in analyses with dementia as outcome (Table S12). Seven, we evaluated the impact of leaving out individual health factors from Life's Essential 8 score on mediation analyses for SEP and dementia. Results showed that leaving out smoking resulted in the strongest reduction in proportion mediation, both in Whitehall II (reduction from 36% to 25%) and in UK Biobank (reduction from 24% to 19%; Table S13). Eight, Life's Essential 8 score mediated 18% of the association between low versus high occupational level and dementia in UK Biobank (Table S14).

**Table 4:** Associations of SEP with Alzheimer's disease and vascular dementia: total, direct, and indirect effects in UK Biobank.

	Alzheimer's Disease	Vascular Dementia
n = 278,215	N cases = 1362	N cases = 536
Low versus high educational level	Hazard Ratio (95% CI)	Hazard Ratio (95% CI)
Total effect	1·64 (1·44, 1·83)	1·84 (1·52, 2·31)
Direct effect	1·59 (1·39, 1·79)	1·50 (1·23, 1·89)
Indirect effect	1·03 (1·00, 1·07)	1·23 (1·18, 1·28)
**% mediation**	8%	41%

The hazard ratios are calculated for low versus high SEP. Covariates entered in all analyses in addition to SEP: age at baseline, sex, and ethnicity. Inverse probability weights were included in the models.

*Abbreviations* CI: Confidence Interval; SEP: Socioeconomic Position (measured using educational level).

## Discussion

The present study has two main findings. One, potentially modifiable Life's Essential 8 score (eating better, physical activity, not smoking, healthy sleep, controlling cholesterol, and managing weight, blood sugar and blood pressure) mediated 36% and 24% of the associations between SEP and incident dementia in Whitehall II (31.7-year median follow-up) and UK Biobank (13.5-year median follow-up). Two, Life's Essential 8 score also mediated associations of SEP with most secondary outcomes, more strongly in Whitehall II than in UK Biobank (i.e., for coronary heart disease [63% versus 40%], and mortality [50% versus 36%], but not stroke [34% versus 50%]). As expected, the proportion mediated by Life's Essential 8 score was greater for cardiovascular outcomes and mortality than for the association between SEP and dementia.

Considering both cohort studies in the analyses, this is the largest study to date on mediation of social inequalities by modifiable cardiometabolic factors in the risk of dementia,^6^, ^7^, ^8^, ^9^ and the 31.7-year median follow-up in the Whitehall II study was longer than in previous studies. Main novelties of the present study are the quantification of mediation using data from midlife to evaluate all eight risk factors included in Life's Essential 8 score (sleep being the most recent addition), and to consider subtypes of dementia (Alzheimer's disease, vascular dementia) in additional analyses. Our findings are generally consistent with findings on mediation analyses of the SES-dementia association in previous studies,^6^, ^7^, ^8^, ^9^ but in three out of five previous studies^6^, ^7^, ^8^ risk factors were assessed at older ages rather than in midlife (at age 65^7^ or after age 70^6^^,^^8^). The feasibility and advantages of modifying risk factors at older ages remains unclear. The long preclinical phase of dementia,^3^ also implies that results from these studies are unlikely to be free from reverse causation bias. A fourth study,^9^ measured vascular risk factors in midlife and the 25% mediation is similar in our study. A fifth study,^26^ assessed three of eight items included in Life's Essential 8 score in midlife and found 9% mediation. Besides the use of two cohort studies in the analyses, confidence in our results also stems from the results on secondary outcomes (stroke,^27^ coronary heart disease,^27^, ^28^, ^29^ and mortality^30^^,^^31^) being similar to previous findings. The added value of this study to previous studies is that mediation by the complete Life's Essential 8 score was investigated. Previous studies considered less health factors.^27^, ^28^, ^29^, ^30^, ^31^

The Lancet 2020 has highlighted the importance of prevention for dementia, concluding that up to 40% of dementia can potentially be prevented via modification of 12 risk factors taken from across the life course (education, hearing impairment, traumatic brain injury, hypertension, excessive alcohol consumption, obesity, smoking, depression, social isolation, physical inactivity, air pollution, and diabetes).^2^ Our focus was not on identifying modifiable risk factors for dementia but rather on estimating the extent to which a tool used to promote cardiovascular health could also be used to address social inequalities in dementia by targeting midlife risk factors.

The association between midlife cardiovascular risk factors and late-onset dementia is biologically plausible. Health behaviors and cardiometabolic risk factors are thought to result in a lower risk of cerebral deterioration and cognitive dysfunction, and, can ultimately prevent or delay the onset of clinical dementia.^32^, ^33^, ^34^, ^35^, ^36^, ^37^, ^38^ Cardiovascular risk factors are suggested to contribute to the pathobiology of dementia via their detrimental effects on the cerebral vasculature.^2^^,^^39^ In our study, modifiable health factors mediated ∼35–63% of social inequalities in risk of stroke and coronary heart disease. In accordance, the proportion mediated by these factors was greater for vascular (∼40%) than Alzheimer's disease (8%) dementia. Non-vascular mechanisms are also likely to be involved.^2^^,^^39^ For example, poor sleep and physical inactivity may reduce the clearance of toxic waste products from the brain, such as amyloid beta, which can predispose to cerebral neurodegeneration.^40^^,^^41^ In addition, certain components of a healthy diet may be favorable for neuronal health.^42^^,^^43^

The proportion of social inequalities in risk of dementia mediated by Life's Essential 8 score was larger in Whitehall II than in UK Biobank. There are several possible explanations: occupational level in Whitehall II is a more comprehensive measure of socioeconomic circumstances than education leading to a stronger association with Life's Essential 8 Score in Whitehall II; the measurement of mediators at about 50 years for all participants, allowing longer exposure duration; and the shorter follow-up in UK Biobank that does not allow sufficient time for dementia onset.

Our findings suggest that public health policies may contribute to reducing risk of dementia at a population level by targeting modifiable health factors among socially disadvantaged group where dementia prevalence is known to be higher. Stronger effects of interventions on lifestyle factors may possibly be expected for vascular dementia than for Alzheimer's disease given our findings. However further study is required for robust conclusions on this matter. Our findings may be particularly relevant for countries that are developing nationwide action plans for the prevention of dementia.^1^ However, further research is warranted to evaluate to which extent dementia risk changes due to interventions on modifiable risk factors. The strengths of associations identified in observational data may differ from effect estimates obtained from randomized controlled trials. Observational studies such as ours do also not allow for inference of causality. Future studies should also investigate how modification of risk factors among individuals with a low SEP can be achieved cost-effectively and implemented at the population level.^44^

This study has important strengths. One, use of data from two independent cohorts provided robust insight, allowing for independent validation of findings.^45^ Two, a range of additional analyses were undertaken, including analyses to quantify the impact of measurement error in the mediator and unmeasured confounders.^24^ Three, the systematic use of inverse probability weighting in all analyses reduced the possibility that associations were spuriously estimated due to selection bias.^46^ Four, both cohort studies have standardised assessment of modifiable risk factors (as Life's Essential 8 score) at a clinical examination and the linkage to health records provides outcome data on all participants.

The study has also limitations. One, the measures of SEP used in this study (education, occupation) may not capture the full extent of possible social economic inequalities, such as inequalities in childhood.^47^ Two, we may have underestimated the proportion mediation due to measurement error in the calculation of Life's Essential 8 score.^11^ Indeed, simulation results showed that lower levels of measurement error in the mediator would result in a higher proportion of associations mediated by Life's Essential 8 score. Three, we cannot exclude the possibility that residual confounding impacted our findings due to unmeasured covariates (e.g., genetic and contextual socioeconomic factors [such as family wealth]^48^ and environmental factors such as air pollution)^45^ but the mediational E-values show that any they would need to be strongly associated with Life's Essential 8 score and the outcomes to nullify observed associations.^24^ We accounted for some genetic variation by adjusting for APOE4 genotype in analyses for dementia and found that this additional adjustment did not importantly impact findings. Other factors that were not accounted for and may affect associations under study are chronic morbidity and mental health. Both these factors may be potential confounders as they are associated with exposure, mediator, and outcomes.^49^^,^^50^ They may also be potential mediators between Life's Essential 8 Score and outcomes. Four, in a subset of individuals from Whitehall II (n = 4661) SEP and Life's Essential 8 Score were assessed concurrently, limiting conclusions on temporality^45^; this was not the case in UK Biobank as educational level was attained before study baseline (minimum age = 40) at measure of Life essential 8.

In conclusion, the results from this study add to the evidence on vascular contribution to dementia.^51^ Using data from two independent cohort studies we found that a quarter to third of the excess risk in dementia in socially disadvantaged groups could be explained by modifiable cardiovascular health factors, assessed by Life's Essential 8 score. In addition, Life's Essential 8 score mediated 34–63% of social inequalities in risk of stroke, coronary heart disease, and mortality. Hence, interventions promoting cardiovascular health factors may importantly contribute to reducing social inequalities in risk of dementia as well as cardiovascular outcomes and mortality.

## Contributors

FCTvdH: formal analysis, data curation, investigation, methodology, writing-original draft and writing-review and editing; LV: methodology and writing-review and editing; AD: data curation methodology, software, and writing-review and editing; ID: writing-review and editing, investigation; BL: writing-review and editing, investigation; SS: funding acquisition, investigation, writing-review, editing, co-supervision; AS-M: conceptualisation, funding acquisition, investigation, supervision, writing-review and editing, project administration. FCTvdH conducted formal analyses and AD accessed and verified data.

## Data sharing statement

Data, protocols, and other metadata of the Whitehall II study are available to the scientific community via the Whitehall II study data sharing portal: https://www.ucl.ac.uk/psychiatry/research/mental-health-older-people/whitehall-ii/data-sharing.

## Declaration of interests

Grants for AS-M: NIH (R01AG056477 and RF1AG062553). Grants for SS: European Research Council (101043884) and Agence Nationale de la recherche (ANR-19-CE36-0004-01). Grants for MK: Wellcome Trust (221854/Z/20/Z), UK Medical Research Council (MR/S011676/1), National Institute on Aging (NIH), US (R01AG056477, R01AG062553), Academy of Finland (350426) and Finnish Foundation for Cardiovascular Research (a86898). All other authors declare no competing interests.
