# Supplementary material for: Role of cardiovascular health factors in mediating social inequalities in the incidence of dementia in the UK: two prospective, population-based cohort studies
Source: eClinicalMedicine. 2024 Mar 14;70:102539. doi: 10.1016/j.eclinm.2024.102539 (PMC10955651; doi:10.1016/j.eclinm.2024.102539)
Supplement: Supplementary data [file mmc1.pdf]

Contents

Summary Box

Supplemental Methods

Supplemental Figures

Figure S1. Median (IQR) scores on Life's Essential 8 in categories of SEP (measured using occupational position) in Whitehall II.

Figure S2. Median (IQR) scores on Life's Essential 8 in categories of SEP (measured using education) in UK Biobank.

Supplemental Tables

Table S1. Calculation of Life's Essential 8 score in Whitehall II and UK Biobank.

Table S2. Definitions of direct, indirect, and total effects in the counterfactual framework.

Table S3. Incidence rate per 1000 person-years of dementia, stroke, coronary heart disease, and mortality across SEP categories.

Table S4. P-for-interaction values to examine differences as a function of sex in the analyses.

Table S5. P-for-interaction values to examine differences as a function of ethnicity in the analyses.

Table S6. Association of SEP with coronary heart disease stratified by sex: total, direct, and indirect effects and the proportion mediated by Life's Essential 8 score.

Table S7. Association of SEP with mortality stratified by sex: total, direct, and indirect effects and the proportion mediated by Life's Essential 8 score.

Table S8. Estimates of total, direct, indirect effects, and the proportion mediation for dementia, stroke, coronary heart disease, and mortality allowing for measurement error in Life's Essential 8 score.

Table S9. Mediational E-values calculated for mediation by Life's Essential 8 score in the associations of SEP with incident dementia, stroke, coronary heart disease, and mortality.

Table S10. Associations of SEP with coronary heart disease and mortality in UK Biobank using Cox regression instead of the accelerated failure time model: total, direct, and indirect effects and the proportion mediated by Life's Essential 8 score.

Table S11. Population attributable risk for low versus high Life's Essential 8 score and incident dementia according to SEP.

Table S12. Association of SEP with dementia, with additional adjustment for APOE4: total, direct, and indirect effects and the proportion mediated by Life's Essential 8 score.

Table S13. Association of SEP with dementia (stroke, coronary heart disease, and mortality): total, direct, and indirect effects and the proportion mediated by Life's Essential 8 score estimated from seven instead of eight factors.

Table S14. Association of occupational position with dementia in UK Biobank: total, direct, and indirect effects and the proportion mediated by Life's Essential 8 score.

Summary box

What is already known?

- Markers of socioeconomic disadvantage, measured using education, occupation, or income, are associated with higher dementia risk.
- Modifiable risk factors that ought to be targeted to reduce this excess risk remain unclear.
- There is consensus on the long (15-20 years) preclinical phase of dementia; the implication being that targeting risk factors at older ages in unlikely to be beneficial.

What does this study add?

- Life's Essential 8 is a comprehensive, standardized tool for the assessment of modifiable cardiovascular health factors, including health behaviours and cardiometabolic risk factors.
- Using two longitudinal studies, Whitehall II and UK Biobank with a median follow-up of 31.7 and 13.5 years respectively, we show that a third of the excess risk of dementia in socially disadvantaged groups can be explained by Life's Essential 8.
- As expected, the contribution of Life's Essential 8 to social inequalities in cardiovascular diseases and total mortality was larger and similar to that in the literature, providing confidence in the findings on dementia.

Supplemental Methods

Assessment of Life's Essential 8 score

Table S1 shows the assessment and calculation of Life's Essential 8 score in Whitehall II and UK Biobank. The score (0-800 points) is calculated by summing scores for individual components (each ranging between 0 and 100 points), and consists of the following items: diet, physical activity, smoking, sleep duration, body mass index, blood lipids, blood glucose, and blood pressure.[1]

The assessment of items used to calculate exposure to risk factors included in Life's Essential 8 score has previously been described in detail,[2-4] here we provide a brief description. Questionnaires were used to assess smoking status (current, former, never), physical activity (minutes per week), medication use, sleep (average hours per night), and dietary intake. As previously reported, [2, 3] in the Whitehall II Study adherence to a healthy diet was assessed from weekly intake of fruit and vegetables; and in UK Biobank adherence to a healthy diet was assessed from nine items (fruit and vegetables; fish, processed meat, red meat, milk, butter, amount of cereal, salt, and water). Body-mass index (kg/m^2) and systolic and diastolic blood pressure (mm Hg) were assessed during a clinical examination. [2, 3] Fasting plasma samples were used to assess cholesterol (total cholesterol [mg/dl] and high-density lipoprotein cholesterol [mg/dl]), fasting plasma glucose (mg/dl), and glycated haemoglobin (HbA1c; %). [2, 3]

Assessment of occupational level in UK Biobank

Occupational level was assessed as a five-level variable using the Standard Occupational Classification 2000, levels are (from high to low): 1. managers and senior officials; 2. professional occupations or association professional and technical occupations; 3. administrative and secretarial occupations or skilled trades occupations; 4. personal service occupations or sales and customer service occupations; and 5. process, plant and machine operatives or elementary occupations.

Evaluation of assumptions

One, to investigate linearity, we compared model fit when SEP or Life's Essential 8 score were entered in the model as a continuous or categorical variables using the Bayesian information criterion, an indicator of goodness of fit.[5] Results showed the associations to be linear for SEP and Life's Essential 8 score irrespective of the outcomes. Two, we checked the proportional hazards assumption based on Schoenfeld residuals and visual inspection of Kaplan-Meier plots.[6] Three, we tested whether the results of counterfactual mediation analyses changed when an exposure-mediator interaction was incorporated in the model. There was no evidence of such an interaction, leading us not to include any exposure-mediator interaction in the analyses.[7]

Inverse probability weighting to account for missing data

There was evidence of potential selection in the study sample as we found the association between SEP and outcomes to be stronger when examined among participants with data on SEP and outcomes as compared to the more selected sample also requiring non-missing data on components of the Life's Essential 8 score. Inverse probability weighting analyses were thus used to account for the impact of missing data.[8] The 'target population' was defined as participants with no dementia, stroke, or coronary heart disease at SEP measurement. In addition, in Whitehall II, participants who died before assessment of Life's Essential 8 score at age 50 (i.e. death before age 59.9) were also excluded from the target population (as these individuals could not be exposed to risk factors at age 50). Then, the probability of non-missing data on Life's Essential 8 score and covariates in the target population (corresponding to being part of the analytic sample) was calculated using a logistic regression adjusted for demographic, cardiovascular and lifestyle factors at baseline, morbidities including dementia, stroke, coronary heart disease, and mortality over follow-up, and stepwise-selected interactions between covariates. In this model, we used multiple imputation (20 datasets) to account for missing data in covariates. The inverse of these probabilities was used to weight results in the main analyses.

We developed the weights using STATA (StataCorp. 2017. Stata Statistical Software: Release 15. College Station, TX: StataCorp LLC) and used these weights in the CMAverse package in R (R version 4.0.3 [2020-10-10], R Foundation for Statistical Computing, Vienna, Austria).[9] We included these weights in both the regression model with the mediator as outcome ("mreg"); and the regression models with dementia, stroke, coronary heart disease, and mortality as outcomes ("yreg").

Accelerated failure time model

When incidence of outcomes was not rare (i.e. ≥10% of individuals), we analysed associations using an accelerated failure time model. If Cox regression analyses are performed for non-rare outcomes the proportion mediation may be overestimated (as hazard ratios are non-collapsible).[7, 10] The accelerated failure time model calculates the risk of survival free of an outcome (rather than the risk of an outcome as in Cox regression).[7, 10] In order to facilitate interpretation of the accelerated time model results, we expressed results of this analysis in the same direction as the results of Cox regression analyses. To do so, we inverted results of the accelerated failure time model analyses, converting results from survival time ratios to failure time ratios. Inversion was done by dividing the value one by the estimate from the accelerated failure time model (for example the Survival Time Ratio of 0.80 obtained from the accelerated failure time model was recalculated to a Failure Time Ratio of 1.25 [calculated as 1/0.80]).

Measurement error

Measurement error may affect the assessment of Life's Essential 8 Score. We used the R command 'cmsens', included in the R package CMaverse,[9] specifying the SIMEX (simulation-extrapolation) approach to simulate differing level of measurement error in the Life's Essential 8 score.[11] This approach consists of starting with the observed data and examining the trend in parameter estimates when measurement error changes from small to severe (reliability ratio or λ from 0.75 to 0.25) and comparing results with the results obtained using observed data (no measurement error, λ=1). The total, direct, indirect effects, proportion mediation are obtained. 95% CI for effects are obtained from 200 bootstrap replications after correction for measurement error.

References

1. Lloyd-Jones DM, Allen NB, Anderson CAM, Black T, Brewer LC, Foraker RE, Grandner MA, Lavretsky H, Perak AM, Sharma G, Rosamond W and American Heart A. Life's Essential 8: Updating and Enhancing the American Heart Association's Construct of Cardiovascular Health: A Presidential Advisory From the American Heart Association. *Circulation*. 2022;146:e18-e43.
2. Sabia S, Fayosse A, Dumurgier J, Schnitzler A, Empana JP, Ebmeier KP, Dugravot A, Kivimaki M and Singh-Manoux A. Association of ideal cardiovascular health at age 50 with incidence of dementia: 25 year follow-up of Whitehall II cohort study. *BMJ*. 2019;366:l4414.
3. Petermann-Rocha F, Deo S, Celis-Morales C, Ho FK, Bahuguna P, McAllister D, Sattar N and Pell JP. An Opportunity for Prevention: Associations Between the Life's Essential 8 Score and Cardiovascular Incidence Using Prospective Data from UK Biobank. *Curr Probl Cardiol*. 2023;48:101540.
4. Sabia S, Fayosse A, Dumurgier J, van Hees VT, Paquet C, Sommerlad A, Kivimaki M, Dugravot A and Singh-Manoux A. Association of sleep duration in middle and old age with incidence of dementia. *Nat Commun*. 2021;12:2289.
5. Vrieze SI. Model selection and psychological theory: a discussion of the differences between the Akaike information criterion (AIC) and the Bayesian information criterion (BIC). *Psychol Methods*. 2012;17:228-43.
6. In J and Lee DK. Survival analysis: part II - applied clinical data analysis. *Korean J Anesthesiol*. 2019;72:441-457.
7. Valeri L and Vanderweele TJ. Mediation analysis allowing for exposure-mediator interactions and causal interpretation: theoretical assumptions and implementation with SAS and SPSS macros. *Psychol Methods*. 2013;18:137-50.
8. Mansournia MA and Altman DG. Inverse probability weighting. *BMJ*. 2016;352:i189.
9. Shi B, Choirat C, Coull BA, VanderWeele TJ and Valeri L. CMAverse: A Suite of Functions for Reproducible Causal Mediation Analyses. *Epidemiology*. 2021;32:e20-e22.
10. VanderWeele TJ and Ding P. Sensitivity Analysis in Observational Research: Introducing the E-Value. *Ann Intern Med*. 2017;167:268-274.
11. Valeri L, Lin X and VanderWeele TJ. Mediation analysis when a continuous mediator is measured with error and the outcome follows a generalized linear model. *Statistics in medicine*. 2014;33:4875-4890.

Supplemental Figures

Figure S1. Median (IQR) scores on Life's Essential 8 in categories of SEP (measured using occupational position) in Whitehall II.

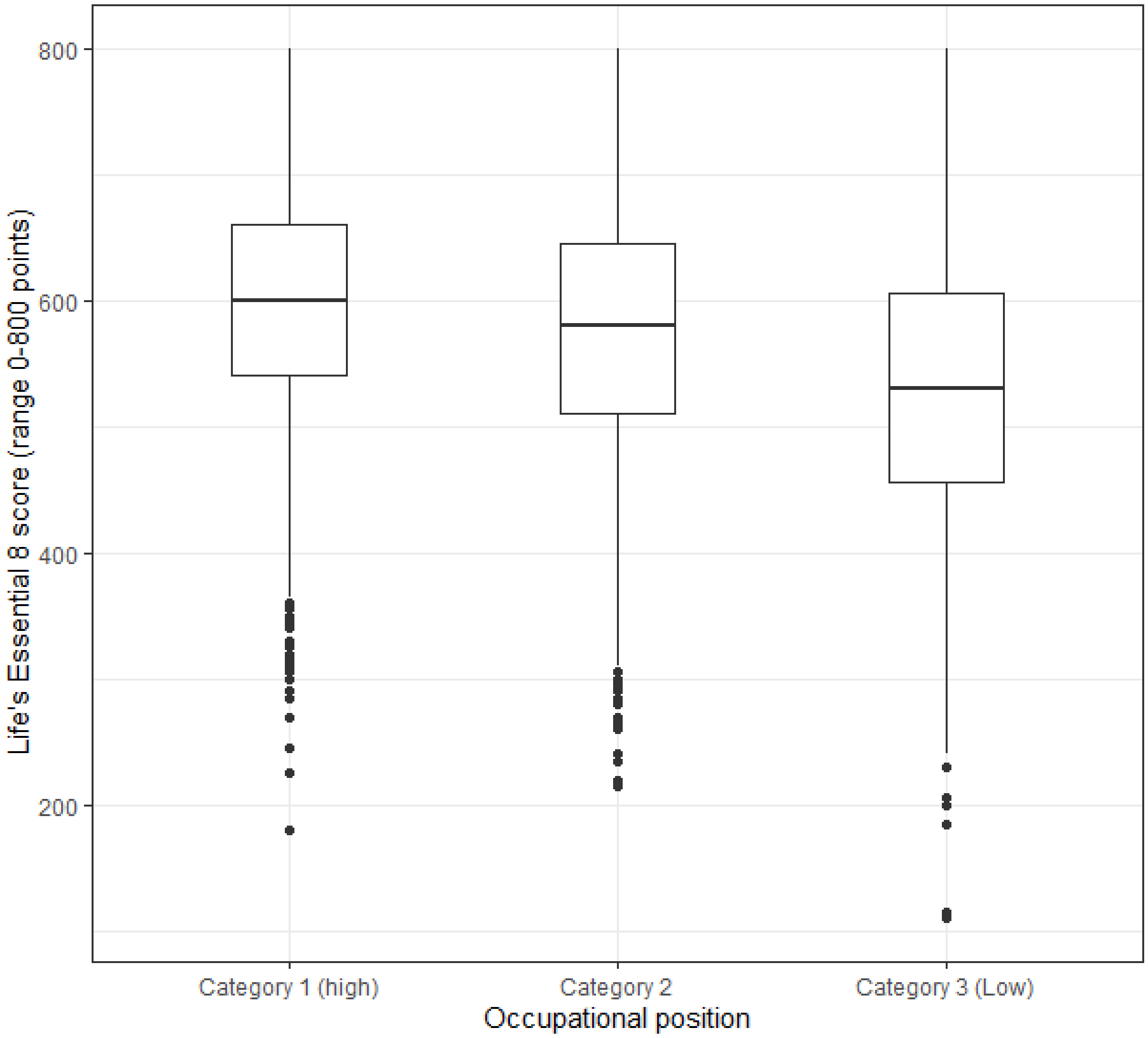

Abbreviations IQR: inter-quartile range; SEP: socioeconomic position.

Figure S2. Median (IQR) scores on Life's Essential 8 in categories of SEP (measured using education) in UK Biobank.

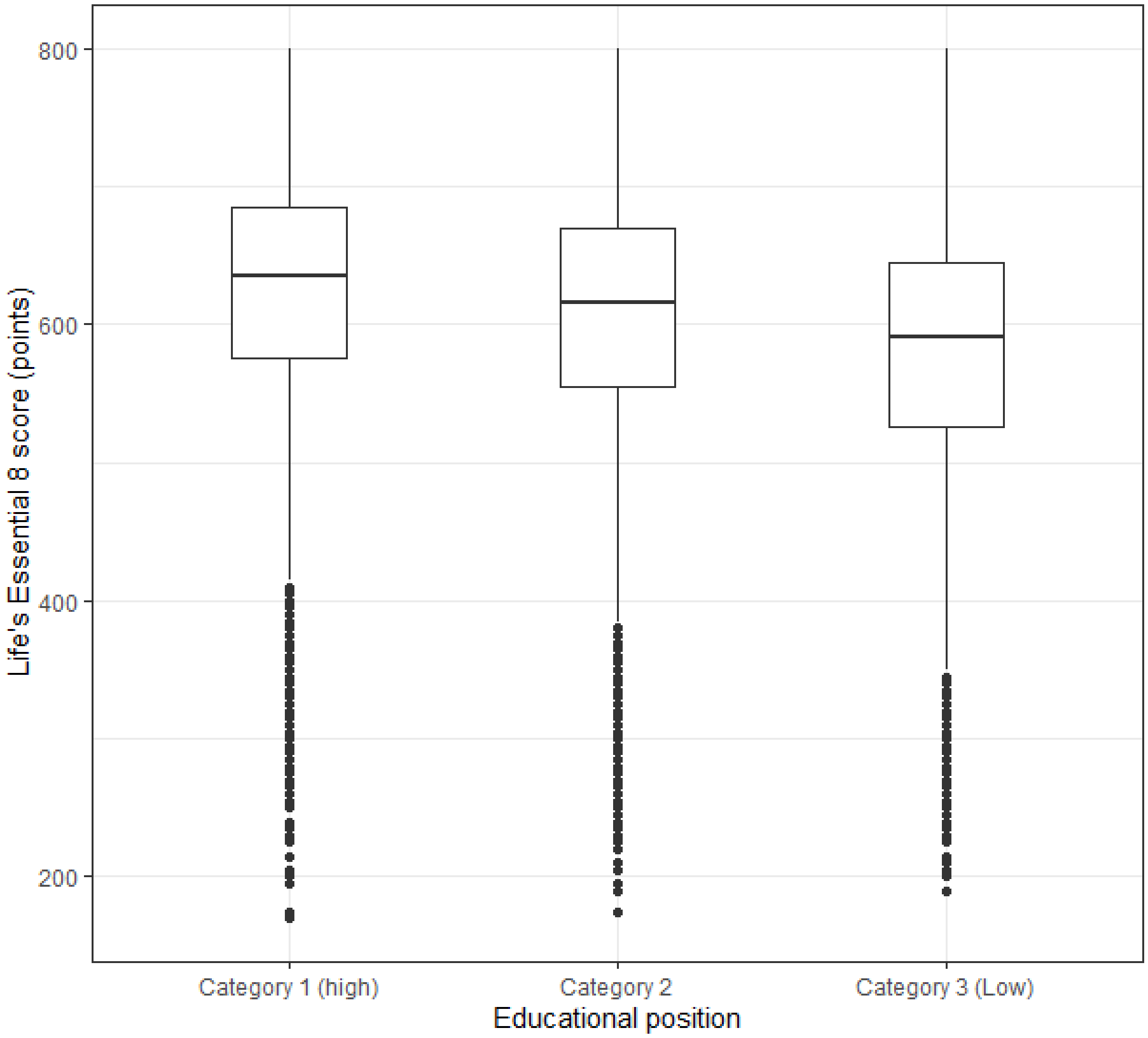

Abbreviations IQR: inter-quartile range; SEP: socioeconomic position.

Supplemental Tables

Table S1. Calculation of Life's Essential 8 score in Whitehall II and UK Biobank.

	Whitehall II			UK Biobank		
**Health Metric**	**Information**	**Scoring**		**Information**	**Scoring**	
		**Points**	**Status**		**Points**	**Status**
**Smoking**	Smoking was classified using self-reported data	100<br>75<br>50<br>25<br>0	Never<br>Previous (quit ≥5 years ago) [1]<br>Previous (quit 1-5 years ago)[1]<br>Previous (quit <1 year ago)[1]<br>Current	Smoking was classified using self-reported data.	100<br>50<br>0	Never<br>Previous<br>Current
**Body mass index**	Body weight (kg) divided by height squared (m^2)	100<br>70<br>30<br>15<br>0	<25 kg/m^2<br>25.0 to 29.9 kg/m^2<br>30.0 to 34.9 kg/m^2<br>35.0 to 39.9 kg/m^2<br>≥40.0 kg/m^2	Body weight (kg) divided by height squared (m^2)	100<br>70<br>30<br>15<br>0	<25 kg/m^2<br>25.0 to 29.9 kg/m^2<br>30.0 to 34.9 kg/m^2<br>35.0 to 39.9 kg/m^2<br>≥40.0 kg/m^2
**Physical activity**	Self-reported moderate or vigorous physical activity	100<br>90<br>80<br>60<br>40<br>20<br>0	≥150 min/week<br>≥120 to <150 min/week<br>≥90 to <120 min/week<br>≥60 to <90 min/week<br>≥30 to <60 min/week<br>>0 to <30 min/week<br>0 min/week	Self-reported physical activity converted to MET/week	100<br>90<br>80<br>60<br>40<br>20<br>0	≥600 MET min/week<br>≥480 to <600 MET min/week<br>≥360 to <480 MET min/week<br>≥240 to <360 MET min/week<br>≥120 to <240 MET min/week<br>≥4 to <120 MET min/week<br>0 MET min/week
**Healthy diet score**	Self-reported fruit and vegetable intake and considered in four groups[2]	100<br><br>65<br>25<br>0	Highest score (score ≥95-100%)<br>3rd quartile (≥50-<95%)<br>2nd quartile (≥25-<50%)<br>Lowest quartile (0-<25%)	Diet score created for UK Biobank and split into five groups	100<br>75<br>50<br>25<br>0	Highest score (score ≥95-100%)<br>4th group (≥75-<95%)<br>3rd group (≥50-<75%)<br>2nd second (≥25-<50%)<br>Lowest quartile (0-<25%)
**Cholesterol**	Non-HDL cholesterol[3] and self-reported use of lipid-lowering medication	100<br><br>50<br><br>0	<130 mg/dl<br>≥130 to 189 mg/dl<br>≥160-189 mg/dl<br>≥190 to 219 mg/dl<br>≥220 mg/dl<br><br>In the case of lipid-lowering medication use 20 points were subtracted from the score	Non-HDL cholesterol and self-reported lipid-lowering medication use	100<br>60<br>40<br>20<br>0	<130 mg/dl<br>≥130 to 189 mg/dl<br>≥160-189 mg/dl<br>≥190 to 219 mg/dl<br>≥220 mg/dl<br><br>In the case of lipid-lowering medication use 20 points were subtracted from the score
**Blood pressure**	Systolic and diastolic blood pressure and self-reported	100<br>75<br>50<br>25	<120 & <80 mmHg<br>≥120 to 129 & <80 mmHg<br>≥130 to 139 or ≥80 to 89 mmHg<br>≥140 to 159 or ≥90 to 99 mmHg	Systolic and diastolic blood pressure and self-reported	100<br>75<br>50<br>25	<120 & <80 mmHg<br>≥120 to 129 & <80 mmHg<br>≥130 to 139 or ≥80 to 89 mmHg<br>≥140 to 159 or ≥90 to 99 mmHg

	antihypertensive medication use	0	≥160 or ≥100 mmHg<br><br>In the case of antihypertensive medication use 20 points were subtracted from the score	antihypertensive medication use	0	≥160 or ≥100 mmHg<br><br>In the case of antihypertensive medication use 20 points were subtracted from the score
**HbA1c**	Diabetes (based on self-report and medication use) and fasting plasma glucose[4]	100<br>60<br><br>20	Without diabetes<br>Fasting plasma glucose ≥100 to 126 mg/dl<br>With diabetes	HbA1c	100<br>60<br>40<br>30<br>20<br>10<br>0	<5.7 %<br>≥5.7 to 6.4 %<br>HbA1c <7.0%<br>HbA1c ≥7.0 to 7.9%<br>HbA1c ≥8.0 to 8.9%<br>HbA1c ≥9.0 to 9.9%<br>HbA1c ≥10.0%
**Sleep**	Self-reported average hours of sleep per night	100<br>70<br>40<br>20	≥7 to <9 h/day<br>≥6 to <7 h/day<br>>9 h/day<br><5 h/day	Self-reported average hours of sleep per night	100<br>90<br>70<br>40<br>20<br>0	≥7 to <9 h/day<br>≥9 to <10 h/day<br>≥6 to <7 h/day<br>≥5 to <6 or ≥10 h/day<br>≥4 to <5 h/day<br><4 h/day

[1]For some participants in Whitehall II data on years since giving up smoking was not available (participants aged 35-59.9 years old in 1991), for these participants no distinction in quitting time was made among former smokers (all provided 50 points)
[2]We used four instead of five categories of fruit and vegetable intake as there was insufficient contrast in intake between the 4th ranked (≥75-<95%; which is suggested to award 75points) and the 5th ranked group (≥50-<75%; which is suggested to award 50 points). Individuals with a score in the 4th and 5th group combined were hence all awarded 65 points.
[3]When data on HDL were not available in the Whitehall II Study we calculated the score for cholesterol based on total cholesterol and use of lipid-lowering medication (instead of non-HDL) to maximize the numbers of individuals that could be included in analyses (0 points: total cholesterol≥240 mg/dl; 50 points: total cholesterol<200mg/dl and use of lipid-lowering medication or total cholesterol 200-239 mg/dl; 100 points: total cholesterol<200 mg/dl; these categories were based on ideal cardiovascular health metrics definitions from the American Heart Association).
[4]Data on fasting plasma glucose were not available at the 1985 wave in the Whitehall II Study, we used information on diabetes status.
Abbreviations HDL: high density lipoprotein; HbA1c: glycated haemoglobin.

Table S2. Definitions of direct, indirect, and total effects in the counterfactual framework.

**Counterfactual effect definition on the Hazard Ratio scale**[a]	
Direct Effect	$\frac{\lambda_{T_{a,M_{a^*}}}(t\vert c)}{\lambda_{T_{a^*,M_{a^*}}}(t\vert c)}$
Indirect Effect	$\frac{\lambda_{T_{a,M_a}}(t\vert c)}{\lambda_{T_{a,M_{a^*}}}(t\vert c)}$
Total Effect	$\frac{\lambda_{T_{a,M_{a^*}}}(t\vert c)}{\lambda_{T_{a^*,M_{a^*}}}(t\vert c)} \times \frac{\lambda_{T_{a,M_a}}(t\vert c)}{\lambda_{T_{a,M_{a^*}}}(t\vert c)}$

[a]An exposure of interest (here socioeconomic position), *T* time-to-event outcome (dementia/stroke/coronary heart disease/death), *M* mediator (Life's Essential 8 score) and *C* set of covariates (age at baseline, sex, and ethnicity). For example, $\lambda_{T_{a,M_{a^*}}}(t|c)$ is the individual's conditional hazard at time *t* if the exposure had been set to *a* and the mediator had been set to the level it would have been had exposure been *a**. Similar definitions can also be drawn for the effects in terms of mean Survival Time Ratios.

Table S3. Incidence rate per 1000 person-years of dementia, stroke, coronary heart disease, and mortality across SEP categories.

SEP category	Dementia	Stroke	Coronary heart disease	Mortality
	**IR (95% CI)**	**IR (95% CI)**	**IR (95% CI)**	**IR (95% CI)**
**Whitehall II Study**				
Occupational position				
1 (high)	1.11 (0.86, 1.43)	0.96 (0.73, 1.27)	3.71 (3.23, 4.26)	4.11 (3.59, 4.71)
2	1.29 (1.05, 1.58)	1.07 (0.85, 1.35)	4.29 (3.82, 4.82)	5.13 (4.60, 5.72)
3 (low)	1.88 (1.57, 2.24)	1.41 (1.14, 1.74)	5.27 (5.73, 5.87)	6.35 (5.75, 7.02)
**UK Biobank**				
Educational level				
1 (high)	0.46 (0.43, 0.49)	0.92 (0.87, 0.97)	3.06 (2.97, 3.14)	2.76 (2.68, 2.84)
2	0.55 (0.51, 0.59)	1.10 (1.04, 1.16)	3.62 (3.51, 3.73)	3.21 (3.11, 3.31)
3 (low)	0.72 (0.66, 0.78)	1.25 (1.16, 1.33)	4.54 (4.38, 4.71)	4.15 (4.00, 4.31)

IR were adjusted for age at baseline, sex, and ethnicity.
Abbreviations IR: incidence rate; CI: confidence interval; SEP: socioeconomic position (measured using occupational position in Whitehall II and using educational level in UK Biobank).

Table S4. P-for-interaction values to examine differences as a function of sex in the analyses.

	Whitehall II Study	UK Biobank
	**P-value**	**P-value**
Low versus high SEP → Life’s Essential 8 Score	0.75	0.07
Life’s Essential 8 score → dementia	0.93	0.22
Low versus high SEP → dementia	0.77	0.13
Life’s Essential 8 score → stroke	0.20	0.35
Low versus high SEP → stroke	0.14	0.41
Life’s Essential 8 score → coronary heart disease	**0.01**	**<0.001**
Low versus high SEP → coronary heart disease	**<0.001**	**<0.001**
Life’s Essential 8 score → mortality	**<0.001**	**0.01**
Low versus high SEP → mortality	0.21	**<0.001**

P-values are shown for the interaction terms of sex with the exposure variable (e.g. SEP*sex) or mediator variable (e.g. sex*Life’s Essential 8 score). Covariates entered in all models in addition to SEP (low versus high) or Life’s Essential 8 score: sex, and ethnicity. Age at baseline was used at the timescale in all analyses. We used Cox regression analyses for dementia and stroke and accelerated failure time model for coronary heart disease and mortality. Inverse probability weights were used in all analyses.
Abbreviations SEP: socioeconomic position (measured using occupational position in Whitehall II and educational level in UK Biobank).

Table S5. P-for-interaction values to examine differences as a function of ethnicity in the analyses.

	Whitehall II Study	UK Biobank
P-values for interaction by ethnicity	**P-value**	**P-value**
Low versus high SEP → Life's Essential 8 Score	0.21	0.02
Life's Essential 8 score → dementia	0.77	0.54
Low versus high SEP → dementia	0.62	0.88

P-values are shown for the interaction terms of ethnicity with the exposure variable (e.g. SEP*ethnicity) or mediator variable (e.g. sex*Life's Essential 8 score). Covariates entered in all models in addition to SEP (low versus high) or Life's Essential 8 score (per 100 points): age at baseline, sex, and ethnicity. Age at baseline was used at the timescale in analyses with dementia as outcome. We used Cox regression analyses. Inverse probability weights were used in all analyses.

Abbreviations SEP: socioeconomic position (measured using occupational position in Whitehall II and educational level in UK Biobank).

Table S6. Association of SEP with coronary heart disease stratified by sex: total, direct, and indirect effects and the proportion mediated by Life's Essential 8 score.

	Men	Women
**Whitehall II Study**	**N= 6,511**<br>**N cases=1,429**	**N= 3,177**<br>**N cases=536**
Low versus high occupational position	**Hazard Ratio (95% CI)**	**Hazard Ratio (95% CI)**
Total effect	1·19 (1·08, 1·33)	1·39 (1·22, 1·69)
Direct effect	1·04 (0·95, 1·16)	1·20 (1·04, 1·49)
Indirect effect	1·15 (1·11, 1·18)	1·15 (1·11, 1·19)
**% mediation**	74%	40%
**UK Biobank**	**N= 148,648**<br>**N cases=7,576**	**N= 129,567**<br>**N cases=14,025**
Low versus high educational level	**Hazard ratio (95% CI)**	**Hazard Ratio (95% CI)**
Total effect	1·43 (1·37, 1·49)	1·35 (1·32, 1·39)
Direct effect	1·22 (1·18, 1·28)	1·18 (1·14, 1·22)
Indirect effect	1·16 (1·15, 1·18)	1·15 (1·14, 1·16)
**% mediation**	39%	41%

Hazard ratios and Failure Time Ratios are calculated for low versus high SEP. Covariates entered in all analyses in addition to SEP: age at baseline, sex, and ethnicity. Inverse probability weights were included in the models.
Abbreviations CI: Confidence Interval; SEP: Socioeconomic Position (measured using occupational position in Whitehall II and educational level in UK Biobank).

Table S7. Association of SEP with mortality stratified by sex: total, direct, and indirect effects and the proportion mediated by Life's Essential 8 score.

	Men	Women
**Whitehall II Study**	**N= 6,511**<br>**N cases=1,300**	**N= 3,177**<br>**N cases=701**
Low versus high occupational position	**Hazard Ratio (95% CI)**	**Hazard Ratio (95% CI)**
Total effect	1·32 (1·22, 1·45)	1·10 (1·00, 1·20)
Direct effect	1·19 (1·10, 1·30)	0·99 (0·90, 1·08)
Indirect effect	1·10 (1·09, 1·12)	1·11 (1·09, 1·14)
**% mediation**	32%	100%
**UK Biobank**	**N= 148,648**<br>**N cases=7,841**	**N= 129,567**<br>**N cases=14,025**
Low versus high educational level	**Hazard ratio (95% CI)**	**Hazard Ratio (95% CI)**
Total effect	1·22 (1·19, 1·28)	1·32 (1·28, 1·37)
Direct effect	1·12 (1·09, 1·16)	1·19 (1·15, 1·22)
Indirect effect	1·09 (1·09, 1·10)	1·11 (1·11, 1·12)
**% mediation**	40%	35%

Hazard ratios and Failure Time Ratios are calculated for low versus high SEP. Covariates entered in all analyses in addition to SEP: age at baseline, sex, and ethnicity. Inverse probability weights were included in the models.

Abbreviations CI: Confidence Interval; SEP: Socioeconomic Position (measured using occupational position in Whitehall II and educational level in UK Biobank).

Table S8. Estimates of total, direct, indirect effects, and the proportion mediation for dementia, stroke, coronary heart disease, and mortality allowing for measurement error in Life's Essential 8 score.

	**Dementia**		**Stroke**		**Coronary heart disease**		**Mortality**	
**Whitehall II Study**	**N cases=606**		**N cases=463**		**N cases=1,965**		**N cases=2,001**	
**Reliability ratio (λ)[a]**	**Hazard Ratio (95% CI)**	**% mediation**	**Hazard Ratio (95% CI)**	**% mediation**	**Failure Time Ratio (95% CI)**	**% mediation**	**Failure Time Ratio (95% CI)**	**% mediation**
**λ = 1 (observed)**								
Total Effect	1·85 (1·42, 2·32)		1·52 (1·07, [illegible]		1·25 (1·15, 1·37)		1·22 (1·16, 1·30)	
Direct Effect	1·54 (1·17, 1·98)		1·34 (0·96, [illegible]		1·08 (0·99, 1·18)		1·10 (1·03, 1·18)	
Indirect Effect	1·20 (1·12, 1·28)	36%	1·13 (1·05, [illegible]	34%	1·16 (1·12, 1·19)	50%	1·11 (1·09, 1·12)	50%
**λ = 0·75**								
Total Effect	1·85 (1·37, 2·38)		1·52 (1·13, [illegible]		1·25 (1·15, 1·37)		1·22 (1·15, 1·31)	
Direct Effect	1·52 (1·17, 1·95)		1·33 (0·98, [illegible]		1·06 (0·98, 1·17)		1·09 (1·04, 1·17)	
Indirect Effect	1·22 (1·14, 1·29)	39%	1·14 (1·05, [illegible]	35%	1·17 (1·14, 1·20)	68%	1·12 (1·10, 1·14)	54%
**λ = 0·50**								
Total Effect	1·85 (1·43, 2·33)		1·52 (1·10, [illegible]		1·25 (1·15, 1·38)		1·22 (1·15, 1·29)	
Direct Effect	1·46 (1·11, 1·86)		1·29 (0·91, [illegible]		1·03 (0·96, 1·14)		1·06 (1·00, 1·14)	
Indirect Effect	1·27 (1·16, 1·38)	46%	1·17 (1·08, [illegible]	44%	1·21 (1·17, 1·24)	84%	1·14 (1·11, 1·17)	66%
**λ = 0·25**								
Total Effect	1·84 (1·45, 2·41)		1·51 (1·20, [illegible]		1·24 (1·13, 1·37)		1·21 (1·15, 1·30)	
Direct Effect	1·38 (1·07, 1·84)		1·24 (0·94, [illegible]		0·99 (0·90, 1·09)		1·03 (0·98, 1·10)	
Indirect Effect	1·33 (1·23, 1·52)	55%	1·22 (1·11, [illegible]	53%	1·26 (1·22, 1·32)	100%	1·18 (1·15, 1·21)	83%
**UK Biobank**	**N cases=4,649**		**N cases=5,840**		**N cases=21,601**		**N cases=19,089**	
**Reliability ratio (λ)[a]**	**Hazard Ratio (95% CI)**	**% mediation**	**Hazard Ratio (95% CI)**	**% mediation**	**Failure Time Ratio (95% CI)**	**% mediation**	**Failure Time Ratio (95% CI)**	**% mediation**
**λ = 1 (observed)**								
Total Effect	1·65 (1·52, 1·78)		1·40 (1·32, [illegible]		1·41 (1·37, 1·45)		1·30 (1·27, 1·33)	
Direct Effect	1·49 (1·37, 1·61)		1·20 (1·12, [illegible]		1·20 (1·18, 1·23)		1·18 (1·15, 1·19)	
Indirect Effect	1·11 (1·09, 1·12)	24%	1·17 (1·15, [illegible]	50%	1·16 (1·14, 1·18)	40%	1·11 (1·10, 1·12)	36%
**λ = 0·75**								
Total Effect	1·65 (1·53, 1·78)		1·40 (1·30, [illegible]		1·41 (1·36, 1·45)		1·30 (1·26, 1·32)	
Direct Effect	1·48 (1·37, 1·60)		1·19 (1·10, [illegible]		1·20 (1·16, 1·23)		1·16 (1·14, 1·19)	
Indirect Effect	1·11 (1·09, 1·13)	26%	1·18 (1·16, [illegible]	53%	1·18 (1·17, 1·18)	43%	1·11 (1·11, 1·12)	39%

**λ = 0·50**								
Total Effect	1·66 (1·53, 1·78)		1·40 (1·31, [illegible]		1·41 (1·37, 1·46)		1·30 (1·28, 1·33)	
Direct Effect	1·46 (1·34, 1·57)		1·16 (1·07, [illegible]		1·17 (1·14, 1·21)		1·14 (1·12, 1·17)	
Indirect Effect	1·14 (1·11, 1·16)	31%	1·21 (1·19, [illegible]	61%	1·21 (1·20, 1·22)	51%	1·14 (1·13, 1·14)	46%
**λ = 0·25**								
Total Effect	1·67 (1·54, 1·80)		1·41 (1·33, [illegible]		1·41 (1·37, 1·46)		1·30 (1·27, 1·33)	
Direct Effect	1·42 (1·32, 1·54)		1·11 (1·05, [illegible]		1·12 (1·08, 1·15)		1·11 (1·14, 1·09)	
Indirect Effect	1·17 (1·14, 1·20)	37%	1·27 (1·24, [illegible]	72%	1·26 (1·25, 1·27)	64%	1·17 (1·16, 1·18)	57%

The hazard ratios are calculated for low versus high SEP.
Measurement error is reflected in the reliability ratio (λ) which is small at λ=0.75 and large when λ=0.25.
Covariates entered in all models: age at baseline, sex, and ethnicity. In addition, inverse probability weights were included in models.
For all analyses n=200 bootstraps were used to calculate 95% confidence intervals, except for analyses with dementia and stroke as outcomes in UK Biobank for which n=50 bootstraps were used to calculate confidence intervals.
Abbreviations CI: confidence interval; SEP: socioeconomic position (measured using occupational position in Whitehall II and educational level in UK Biobank).

Table S9. Mediational E-values calculated for mediation by Life's Essential 8 score in the associations of SEP with incident dementia, stroke, coronary heart disease, and mortality.

	**Dementia**	**Stroke**	**Coronary heart disease**	**Mortality**
	HR required to nullify (HR required to move to statistically non-significant)	HR required to nullify (HR required to move to statistically non-significant)	FTR required to nullify (FTR required to move to statistically non-significant)	FTR required to nullify (FTR required to move to statistically non-significant)
**Whitehall II Study**				
Low versus high occupational position				
E-value	1·69 (1.49)	1·51 (1.28)	1·59 (1.49)	1·46 (1.40)
**UK Biobank**				
Low versus high educational position				
E-value	1·46 (1.40)	1·62 (1.57)	1·45 (1.42)	1·36 (1.34)

The mediational E-value indicates the minimum strength of association that an unmeasured confounder would need to have with both the mediator and the outcome to explain away the observed association.

The mediational E-values are calculated for Life's Essential 8 score using hazard ratios from models that adjusted for age at baseline, sex, and ethnicity. In addition, inverse probability weights were included in the models.

Abbreviations CI: confidence interval; SEP: socioeconomic position (measured using occupational position in Whitehall II and educational level in UK Biobank); HR, hazard ratio; FTR, Failure Time Ratio.

Table S10. Associations of SEP with coronary heart disease and mortality in UK Biobank using Cox regression instead of the accelerated failure time model: total, direct, and indirect effects and the proportion mediated by Life's Essential 8 score.

	Coronary heart disease	Mortality
**UK Biobank, n= 278,215**	**N cases=21,601**	**N cases=19,089**
Low versus high educational level	**Hazard Ratio (95% CI)**	**Hazard Ratio (95% CI)**
Total effect	1·54 (1·49, 1·59)	1·55 (1·50, 1·62)
Direct effect	1·27 (1·23, 1·32)	1·30 (1·26, 1·36)
Indirect effect	1·21 (1·20, 1·22)	1·19 (1·18, 1·20)
% mediation	50%	45%

The hazard ratios are calculated for low versus high SEP. Covariates entered in all analyses in addition to SEP: age at baseline, sex, and ethnicity.
Inverse probability weights were included in the models.
Abbreviations CI: Confidence Interval; SEP: Socioeconomic Position (measured using educational level in UK Biobank).

Table S11. Population attributable risk for low versus high Life's Essential 8 score and incident dementia according to SEP.

**Whitehall II Study, n= 9,688**			
Occupational position	**Hazard Ratio (95% CI)**	**Frequency**	**Population attributable risk**
High	1·60 (1·17, 2·20)	0.40	0·19 (0·06, 0·32)
Middle	1·51 (1·16, 1·96)	0.49	0·20 (0·07, 0·32)
Low	1·60 (1·17, 2·20)	0.66	0·28 (0·10, 0·44)
**UK Biobank, n= 278,215**			
Educational level	**Hazard Ratio (95% CI)**	**Frequency**	**Hazard ratio (95% CI)**
High	1·26 (1·15, 1·38)	0.44	0·10 (0·07, 0·14)
Middle	1·22 (1·09, 1·35)	0.54	0·11 (0·05, 0·16)
Low	1·30 (1·15, 1·47)	0.66	0·17 (0·09, 0·24)

Population attributable risk was calculated for low versus high Life's Essential 8 score and incident dementia according to level of SEP. Life's Essential score was divided into low (<median value) and high (≥median value). Median values were 580 and 620 points in Whitehall II and UK Biobank, respectively. The following formula was used: f(HR–1)/[1+f(HR–1)], where f refers to frequency of low levels of Life's Essential 8 score and HR refers to risk of incident dementia for low versus high Life's essential 8 score.
Abbreviations: HR, hazard ratio; CI: Confidence Interval; SEP: Socioeconomic Position (measured using occupational position in Whitehall II and educational level in UK Biobank).

Table S12. Association of SEP with dementia, with additional adjustment for APOE4: total, direct, and indirect effects and the proportion mediated by Life's Essential 8 score.

	**Model without APOE4**	**Model with APOE4**
**Whitehall II Study, n= 5,855**	**N cases=339**	**N cases=339**
Low versus high occupational position	**Hazard Ratio (95% CI)**	**Hazard Ratio (95% CI)**
Total effect	1·88 (1·32, 2·67)	2·00 (1·37, 2·89)
Direct effect	1·61 (1·16, 2·36)	1·73 (1·20, 2·49)
Indirect effect	1·16 (1·09, 1·25)	1·16 (1·07, 1·24)
**% mediation**	30%	27%
**UK Biobank, n= 234,298**	**N cases=3,858**	**N cases=3,858**
Low versus high educational level	**Hazard ratio (95% CI)**	**Hazard Ratio (95% CI)**
Total effect	1·53 (1·40, 1·65)	1·53 (1·40, 1·64)
Direct effect	1·40 (1·29, 1·52)	1·40 (1·28, 1·50)
Indirect effect	1·09 (1·07, 1·11)	1·09 (1·08, 1·11)
**% mediation**	24%	25%

Hazard ratios are calculated for low versus high SEP. Covariates entered in all analyses in addition to SEP: age at baseline, sex, ethnicity and APOE4 (only in specified model).

Abbreviations APOE4, apolipoprotein E4; CI: Confidence Interval; SEP: Socioeconomic Position (measured using occupational position in Whitehall II and educational level in UK Biobank).

Table S13. Association of SEP with dementia (stroke, coronary heart disease, and mortality): total, direct, and indirect effects and the proportion mediated by Life's Essential 8 score estimated from seven instead of eight factors.

	Whitehall II Study, n= 9,688 N cases=606		UK Biobank, n= 278,215 N cases=4,649	
**Life's Essential 8 score**	**Hazard Ratio (95% CI)**	**% mediation**	**Hazard Ratio (95% CI)**	**% mediation**
**Without physical activity**				
Total Effect	1·86 (1·45, 2·46)		1·65 (1·53, 1·77)	
Direct Effect	1·59 (1·22, 2·10)		1·50 (1·38, 1·60)	
Indirect Effect	1·17 (1·11, 1·24)	31%	1·10 (1·08, 1·12)	24%
**Without smoking**				
Total Effect	1·84 (1·36, 2·43)		1·64 (1·54, 1·78)	
Direct Effect	1·63 (1·20, 2·13)		1·52 (1·42, 1·65)	
Indirect Effect	1·13 (1·08, 1·19)	25%	1·08 (1·06, 1·09)	19%
**Without sleep**				
Total Effect	1·85 (1·38, 2·43)		1·65 (1·54, 1·75)	
Direct Effect	1·56 (1·19, 2·03)		1·52 (1·42, 1·62)	
Indirect Effect	1·19 (1·12, 1·26)	34%	1·08 (1·07, 1·10)	20%
**Without body-mass index**				
Total Effect	1·86 (1·44, 2·45)		1·65 (1·54, 1·78)	
Direct Effect	1·56 (1·18, 2·09)		1·49 (1·39, 1·60)	
Indirect Effect	1·19 (1·12, 1·26)	35%	1·11 (1·10, 1·13)	26%
**Without lipid profile**				
Total Effect	1·85 (1·47, 2·43)		1·65 (1·54, 1·78)	
Direct Effect	1·49 (1·17, 2·00)		1·50 (1·39, 1·62)	
Indirect Effect	1·24 (1·16, 1·31)	42%	1·10 (1·08, 1·12)	23%
**Without blood pressure**				
Total Effect	1·84 (1·42, 2·49)		1·65 (1·53, 1·81)	
Direct Effect	1·56 (1·21, 2·09)		1·50 (1·39, 1·63)	
Indirect Effect	1·19 (1·13, 1·27)	34%	1·10 (1·08, 1·11)	23%
**Without diabetes**				
Total Effect	1·85 (1·42, 2·39)		1·65 (1·53, 1·78)	
Direct Effect	1·55 (1·17, 2·06)		1·51 (1·40, 1·64)	
Indirect Effect	1·19 (1·12, 1·27)	35%	1·09 (1·07, 1·11)	21%
**Without diet**				
Total Effect	1·83 (1·41, 2·32)		1·65 (1·52, 1·75)	
Direct Effect	1·60 (1·24, 2·00)		1·50 (1·40, 1·60)	
Indirect Effect	1·15 (1·09, 1·20)	28%	1·10 (1·09, 1·12)	23%

Hazard ratios are calculated for low versus high SEP. Covariates entered in all analyses in addition to SEP: age at baseline, sex, and ethnicity. Inverse probability weights were included in the models.

Abbreviations CI: Confidence Interval; SEP: Socioeconomic Position (measured using occupational position in Whitehall II and educational level in UK Biobank).

Table S14. Association of occupational position with dementia in UK Biobank: total, direct, and indirect effects and the proportion mediated by Life's Essential 8 score.

	Dementia
**UK Biobank, n= 190,505**	**N cases=1,767**
Low versus high occupational level	**Hazard ratio (95% CI)**
Total effect	1·46 (1·24, 1·69)
Direct effect	1·37 (1·17, 1·59)
Indirect effect	1·06 (1·04, 1·08)
**% mediation**	18%

Hazard ratios were calculated for low versus high SEP. Covariates entered in all analyses in addition to SEP: age at baseline, sex, and ethnicity. The five-level occupational position variable was coded as "1" for low occupational level and "0" for high occupational level, with in-between categories coded "0.25", "0.5", and "0.75".
Abbreviations CI: Confidence Interval; SEP: Socioeconomic Position (measured using occupational position).
